# Supplementary material for: Feasibility of SMS booster for alcohol reduction in injury patients in Tanzania
Source: PLOS Glob Public Health. 2022 Nov 29;2(11):e0000410. doi: 10.1371/journal.pgph.0000410 (PMC10022354; doi:10.1371/journal.pgph.0000410)
Supplement: Attachment — Submitted filename: SMS Feasibility Rebuttal Letter_tosubmit.pdf [file pgph.0000410.s002.pdf]

Reviewer #1: This is an interesting manuscript describing a feasibility study of a brief intervention supplemented by SMS boosters, with the aim of reducing alcohol use among persons attending the injury ward with harmful and hazardous alcohol use at admission, in Moshi, Tanzania. The study addresses a very important problem, targets a potentially teachable population, and includes leveraging the power of SMS to potentially increase the efficacy of the in-person intervention. Overall, the methods are sound and the writing is clear. However, the complex study design is a bit confusing and detracts from the presentation, and there are some missing pieces of information which detract from the conclusions. Some specific comments follow.

Thank you very much for your feedback, and the opportunity to revise this manuscript.

1. Overall: the presentation of the results using the checklist framework is novel, however it makes the paper hard to read. It seems that the results are a combination of intervention development and feasibility combined. I think perhaps the whole paper would be easier to read if these were clearly separated. Feasibility seems most relevant after the intervention has been completely developed, i.e. the kinks of sending the messages etc. are worked out.

Thank you for this important feedback. We have worked on separating out the intervention and feasibility components (and delineated sections further in Table 1, and through sub-headings in the Results), and removing the trial description to focus on the SMS alone.

2. Line 130 – is there a reference for the description of the local adaptation?

Thank you; yes, we have added in a reference on line 129.

3. Line 166 – it is not described when and by whom the AUDIT was administered. Also the measure of self-reported alcohol use prior to injury is reported but methods of ascertainment not described.

Thank you. The AUDIT was administered during the registry process, by either a research assistant or research nurse depending on availability. Self-reported alcohol use also occurred during the registry/initial screening. We have added this on lines 165-167.

4. Lines 172-186 and Figure 1. The adaptive RCT design is confusing. It is not clear why different arms are dropped at different stages, except for the reason given for Stage 3. Either more or less detail is needed. For more detail, I wondered how the stages occurred over time, and again, what the decision points were. Figure 1 does not depict the design, just the final allocation, which somehow made me feel like the authors felt the adaptive piece was not relevant which would argue for simplifying the text.

Thank you very much for this important feedback. To ease readability, we have removed the trial portion of the study design, focusing instead on the booster period only. We have added in an

updated study/workflow diagram that shows how boosters were developed and deployed. In addition, we have re-organized our Results around SMS System Implementation and then Feasibility. We hope that this eases understanding of our findings on booster feasibility.

5. There is no description of the BNI and boosters. These do not need to be lengthy, but do need to be included.

Thank you, we have added this into lines 172-178.

6. It is confusing that the paper says its focus is on the SMS portion of the intervention, after describing the complex study design. Results for the entire group are given throughout the paper.

Thank you very much for this important feedback. As mentioned in response to Comment 4, we have removed the trial portion of the study design. Results are only reported according to those given the standard or personalized booster.

7. There is high completion rate so Table 2b is not needed.

Thank you for this comment. We have removed the extraneous table.

8. Lines 242-244. The paper states that phones had to be on and in cell service range to receive messages, but my understanding is that messages will be sent later if a phone is off or out of range. This is suggested by line 258 and lines 264-266 and should be clarified in the methods.

Thank you for this request. Cell phones had to be on and in range for participants to receive messages; otherwise, messages were re-attempted. We hope that lines 189-190 in the Methods and lines 278-281 in the Results clarify this.

9. Were there any safeguards in place to ensure that the intended recipient got the message, rather than others with whom phones may be shared? PINs have been recommended.

Thank you for this important comment, and we acknowledge that that is a concern, as not everyone has their own phone (i.e phone sharing) and info in this text could put health info at risk. During informed consent, however, we noted to patients that for this phone number they are providing, we will be potentially sending texts, and this is the kind of information in them; will you feel comfortable receiving this info on this phone number? They were then given the ability to say yes or no. Sometimes we would get a message back (why are you texting this number, et cetera) - this could be due to a person who owns the phone that we don’t know (i.e, that isn’t our participant), or our participant forgot or doesn't understand. As a safeguard, we call the participant and say this phone number is listed as study participant, can you talk to this person about receiving messages at this phone #, we would be happy to change to another one. We

allow patients therefore to stop info from coming to that number, at any time. It is inherent in the system (since sharing of phones is possible and likely) that info might be shared; so, we have chosen language that even though they talk about alcohol use, is not done in a manner that displays private info. We used this wording to reduce potential harms by the dissemination of info, as we understand there is phone sharing. We hope that lines 367-377 in the Discussion clarify this.

10. Lines 249-255 The paper states that 492 total messages were intended, but only 258 were attempted. There is no discussion of the 234 messages not attempted – why weren't they attempted?

Thank you for this question. The majority of unattempted messages were not transmitted due to errors in the programming of the SMS system; we have added this information into lines 267-269.

11. Lines 271-272 and Table 3 and Table 4a. The observation that SMS delivery during the phases of the study is not helpful given that the reader is not told what measures were taken across phases, and what the phases really were exactly, other than changes in allocations to study arms.

Thank you; we have removed this statement.

12. Line 288 and Table. 5. Six reasons are mentioned but only 3 given in the table. Give all 6.

Thank you, we have included the remaining three in the table.

13. Lines 324-327 are repeats of lines 198-200.

Our sincere apologies for this error, we have removed the repeated lines.

14. Lines 330-337. It says 33 people agreed to answer questions but only 11 responded? Why didn't the other 22 answer the question? Wouldn't the non-response impact interpretation of the results?

Thank you for this question; we are unsure of why the other 22 did not answer the question. We understand that non-response can impact result interpretation;, we believe that the 11 responses are still informative of booster acceptability.

15. Line 350 – says nurse-led BNI – really not described prior. Should be in the methods.

Thank you, we have added this into lines 166-169 and 183-184.

16. Line 357 – 73% success rate for SMS – not sure where this number comes from as the results are hard to read.

Thank you for this feedback. Under “WHEN and HOW MUCH”, 73% comes from the 183 successfully delivered messages under the 251 attempted that were not excluded. We have worked on cleaning this section, and streamlining the study to focus just on the boosters rather than the full PPKAY trial, to ease readability of the Results.

17. Line 358 – “secure system is in place”. I didn’t see this described elsewhere. What does this mean?

Thank you for this feedback. We hope that lines 344-346 in the Discussion clarify this.

Reviewer #2: This paper looked at the feasibility of using SMS messaging as part of an alcohol harm reduction intervention following injury in Tanzania. This is an interesting paper on a very important topic and the feasibility of the intervention was well described within TIDier checklist framework

Thank you as well for your feedback!

1. Due to the Small n in some places there are very small numbers in each cell of the table – where there are less than 3 participants per cell suggest suppressing data to limit potential identification of participants.

Thank you for this comment; we have combined small numbers together in Table 2 to limit potential identification.

2. Table 4- these tables were hard to follow in terms of referring to stage and phase of the study – it would be worth reviewing these and revising the labelling.

Thank you for this comment. We have re-ordered the Results to hopefully help with this issue.

3. Table 6 – it was not clear why some participants did not receive the intervention they were assigned to.

Thank you for this feedback. Here, we are testing the intervention process and differences between standardized and personalized boosters. We aimed to understand the work burden for the study team to do both; the literature indicates that personalized is better, but our knowledge of the workload that requires makes it almost impossible to do everything personalized. Some participants not receiving their allotted intervention could indicate that work burden impacted the fidelity of the arms, in that some received texts when not supposed to, and that some allotted to standard received personalized and vice versa.
