# Supplementary material for: Feasibility of SMS booster for alcohol reduction in injury patients in Tanzania
Source: PLOS Glob Public Health. 2022 Nov 29;2(11):e0000410. doi: 10.1371/journal.pgph.0000410 (PMC10022354; doi:10.1371/journal.pgph.0000410)
Supplement: Attachment — Submitted filename: Response to Reviewers 27July2022.docx [file pgph.0000410.s003.docx]

To PLoS Global Public Health Editors,

Thank you so much for the opportunity to revise this manuscript. As instructed we have created a point by point rebuttal to each editor and reviewer point, a marked up copy and an unmarked up version.

Journal Requirements:

1. Please amend your online detailed Financial Disclosure statement. This is published with the article, therefore should be completed in full sentences and contain the exact wording you wish to be published.

State what role the funders took in the study. If the funders had no role in your study, please state: “The funders had no role in study design, data collection and analysis, decision to publish, or preparation of the manuscript.”

**This was added to the funding disclosure.**

2. Please update your online Competing Interests statement. If you have no competing interests to declare, please state: “The authors have declared that no competing interests exist.”

**This was updated on the online competing interests statement.**

3. Please review your reference list to ensure that it is complete and correct. If you have cited papers that have been retracted, please include the rationale for doing so in the manuscript text, or remove these references and replace them with relevant current references. Any changes to the reference list should be mentioned in the rebuttal letter that accompanies your revised manuscript. If you need to cite a retracted article, indicate the article’s retracted status in the References list and also include a citation and full reference for the retraction notice.

**This has been completed.**

Reviewer Comments:

Reviewer #1: This continues to be an interesting manuscript. The authors have addressed my prior comments but there are a few remaining issues.

1. Line 52 – include “negotiated” in the spelling out of BNI

**Changed.**

2. Lines 65 and 66 and elsewhere: The term “supposed to” sounds unscientific. Replace with crisper terminology?

**Changed throughout.**

3. Line 71: include the word “interviewed”, since the majority were not interviewed

**Changed.**

4. Line 172: I believe accentuate is meant instead of attenuate

**Changed to prolong.**

5. Lines 241-249. How were the messages developed?

**Great question. The process of developing the messages is a whole other ongoing nearly published manuscript. It is outside the scope of this manuscript because the depth and process of adaptation that was required for this content development. As such, we have included this explanation and a very brief overview of the process in the TAILORING section in the results.**

6. Tables 3, 4a, 4b: Since the stages (or phases) are no longer described (which was helpful), the phases and stages listed in these tables are hard to interpret. I know the point is that the delivery improved throughout the study, but maybe this can be said more simply (the extra detail in the tables is not really needed).

**Stages are currently referenced in the text, though with minimal emphasis and details, as previously suggested. Thus, the authors have included all rows on Tables 3 and 4a. We contend that highlighting the differences between stages reveals that stages involved an increasing number of messages and level of intervention complexity, and relative improvement was achieved as the study progressed through each stage. Clarification was added by referring to stages or phases only as “stages” to reduce confusion.**

7. Table 4a: Too many digits in the %s given

**Changed as requested in the table and throughout the manuscript.**

8. Line 297: describe what the quality improvement entailed

**Information was included about this process.**

9. Lines 306-309 and Table 6: Since the focus changed to only the SMS part of the study, data on the other arms are not needed.

**If we understand your suggestion correctly, Table 6 is really demonstrating the complexity of implementation of each arm (not specifically the phase) is not impacting the appropriateness of the intervention fidelity and as such, it is important to include. We have changed it to the type of booster administered compared to no booster intervention to make it make more sense, but we feel that this should be included.**

10. Line 331: Drop “only”, it is editorializing

**Removed.**

11. Lines 344-346: I do think drinking goals is private health information, so I would not assert no info could be shared.

**While drinking goals might not be specific PHI or privileged health information by US standards, the message content at hand may include potentially private health information which could be stigmatizing or information patients don’t wish to tell family or others about. This concern about information being ‘shared’ in the setting of shared cell phones in Tanzania is a real risk voiced by both local collaborators as well as by our local Tanzanian and international regulatory bodies. Our patients gave our feedback that this was, however, not a concern, which is helpful in allaying collaborators' concerns.**

12. Lines 362-364: Not a sentence.

**Good catch, this was edited to be a sentence.**

13. Lines 372-373: I don’t agree with this statement, see #11.

**We have reworded this statement to be more clear; we agree with you. We found that our population was not worried about information security with this text information as stated in the paper-- a concern of others and the regulatory bodies when doing the study. This information is important to dissemination so that we can reassure these concerned parties that this is not a concern.**

14. Lines 373-377: Are you suggesting that family members intercepted the messages? It sounds odd and not related to data presented in the MS.

**Yes. There are situations where family members might have intercepted the messages. Actually we disagree that this is not related to the data presented in the manuscript. This is a feasibility of using text based boosters, and intervention fidelity-- or the intended intervention reaching the person for whom it was intended is a part of that fidelity. If no participant ever received the text, there would be no chance of effectiveness. As such, we believe this is a culturally important process to consider.**

15. Lines 395-397: I think you are talking about the missing 22 interviews. Be explicit that you don’t know why they were not completed and that could have caused bias.

**This was changed as requested.**

Reviewer #3: 1. In the opening sentence, suggested to replace ‘is the leading cause of death’ with ‘contributes to health conditions that cause more than 60% of deaths.

**Changed as requested.**

2. To a reader who is not familiar with the BNI approach, more details need to be provided on how it works and why was it the most appropriate technique for this particular study.

**We have added some information in the introduction about the prevalence of patient level interventions in SSA. Yet, this manuscript is more focused on the text based booster rather than the BNI itself. As such, we have not gone into too much about that portion of the intervention as it is further described in other manuscripts discussing its cultural adaptation etc.**

3. The BNI may not be a reliable technique for arriving at a conclusion regarding the feasibility of an intervention that takes longer (let say, than a month, two, or three). It becomes even complex when the patient has to recall the changes that have taken place over an extended period of time.

**We completely agree. Our BNI is simply the first part of our intervention which is our introduction to our text based boosters which are hopefully going to extend the impact of the BNI. That being said, this is a shorter term impact demonstrating effectiveness at 3 or 6 months. That being said, this is a feasibility study focusing on if a trial using SMS boosters can be performed with fidelity with a short (aka 3 month endpoint).**

4. In the TIDieR framework, the ‘WHAT’ item is limited to ‘materials needed’ to deliver the intervention. What about the actual content of the SMSs? (consider how it affects responses).

**This is a great comment. The process of developing the messages is a whole other ongoing nearly published manuscript. It is outside the scope of this manuscript because the depth and process of adaptation that was required for this content development. As such, we have included this explanation and a very brief overview of the process in the TAILORING section in the results.**

5. In the case of acceptability, if there was no a continuous monitoring of patients’ response as soon as they received the messages, how does one establish (if not informed by a feedback over a prolonged time) that they (participants) ‘enjoyed’ the messages?

**Great point. Acceptability was determined by 1) did they text or call us to stop receiving the messages and 2) did they at the end of follow up inform us that they received the messages and the specifics about that process. The content of the messages which we did not expect to be remembered 6 months later, underwent a completely different extensive development process not described here but in another manuscript given the scope of work required. As such, our acceptability is the acceptability of receiving the information and ‘booster’ by text not the content or remembering the content process.**

7. In the findings, why is fidelity limited to receiving the messages. Were all planned activities implemented as planned? Consider the timing, techniques, etc. In most cases, on the side of the study and study team and not just on the patients. Was there a delivery confirmation strategy? Why didn’t it work? In well-planned interventions, reasons for deviating from plans and milestones are important. Were there any monitoring, evaluation, and learning plans and strategies?

**In the case of our study, feasibility was considered an assessment and faithfully implemented intervention. As such, measures of fidelity (including timing of messages “WHEN”, type of intervention “WHAT”) are included under the category of “Feasibility.” Other strategies of measuring fidelity, such as a delivery confirmation, were not employed in this pragmatic feasibility trial.**

**Your point regarding monitoring is well received. The investigators learned that a systematic process for monitoring fidelity and quality control was lacking. This is referenced in the Discussion section, and detailed in the current revision. An important result of this feasibility study was the subsequent development of a strategy for quality control and intervention monitoring for the full trial. The improved processes include oversight of personalized message content review, weekly review of message delivery, review of unsolicited incoming messages received from participants, and protocols for rectifying and documenting deviations.**

8. There is a contradiction between the information provided in the abstract, methods, findings, and limitations’ sections regarding the number of participants who received a full and intended intervention package in terms of SMSs. Is the correct number 38 or 1?

**Thank you for finding this inconsistency. Of the 41 study participants who were assigned to receive an SMS 38 received them, 3 did not and 1 (outside of the 41) received who wasn’t supposed to. THese numbers were changed in the Abstract and matches the findings.**

9. The conclusion needs to provide key ‘take-home’ lessons regarding the feasibility, the forces that affect the intervention in both positive and negative ways.

**The largest take home message that we added to the conclusion was the need for a rigid yet reactive quality improvement process to ensure SMS fidelity which had been discussed in the discussion.**
